# Does Confucian culture affect public service motivation of grassroots public servants? Evidence from China

**DOI:** 10.3389/fpsyg.2022.1066624

**Published:** 2023-01-18

**Authors:** Cheche Duan, Jiaxin Jiao, Chunzhen Zhao, Yingying Li

**Affiliations:** ^1^School of Government, Shenzhen University, Shenzhen, China; ^2^School of Marxism, Xiamen University, Xiamen, China; ^3^School of Media and Communication, Shenzhen University, Shenzhen, China; ^4^School of Marxism Studies, Zhejiang University of Finance and Economics, Hangzhou, China; ^5^Shenzhen Bao’an International Airport, Shenzhen, China

**Keywords:** Confucian culture, public service motivation, paternalistic leadership, public servants, traditionality

## Abstract

Public service motivation contains distinctive cultural characteristics. Different cultural backgrounds shape public service motives with different connotations and levels. However, the traditional cultural values rooted in historical development and socialization process have not received enough attention in the research on public service motivation. In order to investigate the influence of Confucian culture based on Chinese scenes on public service motivation, in the current study we collected 1308 representative questionnaires from 12 cities in central and eastern China, and adopted the dual fixed effect model and moderating effect model to verify six hypotheses. The empirical results showed that Confucian culture has different effects on public service motivation from four dimensions, namely, attraction to politics and policy making (APP), commitment to public interest (CPI), compassion (COM), and self-sacrifice (SS). The paternalistic leadership plays a part in moderating the influence of Confucian culture on public service motivation. This study not only expands the cross-cultural applicability of the theory of public service motivation in non-western countries, but also supplements the evidence of research on public service motivation in East Asian countries. In practice, it is necessary for the organizations to consider the importance of specific cultural values for organizational culture and personal value orientation.

## 1. Introduction

How to motivate public servants has been a topic of close concern of the public sectors and endless debate among scholars. The emergence of the theory of Public Service Motivation (PSM) provides a new solution to this question. Although public service motivation has profound connotation, the academic community has reached a basic consensus on this concept, that is, the motivation and action aiming at benefiting others and shaping social welfare (Perry and Hondeghem, 2008). Such “altruistic” motivation can stimulate individuals to take action for the interests of a wider range of political organizations at the right time (Vandenabeele, 2007). In understanding the antecedent variables of public service motivation, scholars have noticed that cultural values have an impact on work motivation (Lyons et al., 2006). In fact, the public service motivation reflects a value norm. However, the traditional cultural values rooted in historical development and socialization process have not received enough attention in the research on public service motivation.

In Chinese society, Confucian culture is the most influential traditional culture, and also a key factor in shaping the political attitude of Chinese people (Zhai, 2018). The influence of Confucian culture on Chinese bureaucratic behavior is far-reaching and lasting, and it has spread to East Asian countries and regions. Under the influence of Confucianism, compared with Westerners who place more emphasis on liberalism and individualism, East Asians tend to be more obedient and pay more attention to collectivism (Van der Wal and Yang, 2015), which can improve the organizational performance of Chinese public sectors (Park and Word, 2012). To sum up, Confucian culture may have an impact on the public service motivation of public servants.

Although the literature has touched on the practice and application of Confucian culture in the field of politics and public management, few studies have deeply explored the mechanism between Confucian culture and public service motivation. To what extent is the public service motivation in the Chinese context influenced by Confucian culture? Currently, there is still a lack of serious research and discussion on this question. In view of this, this study attempts to build a theoretical model between traditional Chinese Confucian culture and public service motivation.

Different from previous studies, this study takes Confucian culture as the antecedent variable, and proposes a new theoretical perspective on public service motivation. Based on the empirical data of grassroots public servants in 12 cities in China, and in the real scene of the value field, this study tests the applicability of public service motivation in the Chinese social and cultural background in a bid to expand the explanatory power of the theory of public service motivation in cross-cultural management situations. Additionally, this study adds paternalistic leadership shaped by Confucian culture as a moderating variable to further understand the effect of Confucian culture in different roles and scenes on public service motivation. Furthermore, from the perspective of organizational management, this study suggests that the relationship between culture values and public service motivation should be considered in respect of incentives for public servants in the public personnel management. This study is contributive to the Chinese public sectors to adopt appropriate organizational culture and Confucian leadership to encourage grassroots public servants to continuously engage in the public endeavor of “serving the people wholeheartedly.”

## 2. Literature review and research hypotheses

### 2.1. Understand the principle of public service motivation

#### 2.1.1. Self-determination theory

Self-Determination Theory (SDT) is a motivation theory about human behavior and personality development, which emphasizes the role of matching individual needs with environment (Deci and Ryan, 2008). According to the continuum of motivation, it can be divided into intrinsic motivation, extrinsic motivation and amotivation, in which, extrinsic motivation can be divided into external regulation, introjected regulation, identified regulation, and integrated regulation (Ryan and Deci, 2009, 2017, 2020). SDT is a psychological analysis framework for studying PSM (Ryan and Deci, 2017). Under this framework, some scholars measure the internal motivation of PSM with the “service motivation” of value internalization (Vandenabeele, 2007; Park and Word, 2012; Chen and Bozeman, 2013), and believe that cultural values are the internal factors to stimulate public service motivation (Andrews, 2016). It explains the relationship between cultural values and public service motivation as a whole (Vandenabeele, 2007, 2014; Kim, 2009). From the perspective of self-determinism, some scholars also propose that the inward adjustment of Oriental traditional culture constitutes the identification adjustment of extrinsic motivation for the work values of Chinese civil servants (Han and Sun, 2022). Other scholars argue that public service motivation lies between intrinsic motivation and extrinsic motivation (Jacobsen et al., 2014), or it is regard as the integration of intrinsic motivation and extrinsic motivation. For example, (Breaugh et al., 2018), believe that the rational self-interest dimension of public service motivation is more consistent with external motivation, and altruistic motivation is more consistent with internal motivation. This integrated motivation includes a broad understanding of values and beliefs. In general, SDT provides an effective way to explain why cultural values become the antecedent of public service motivation.

#### 2.1.2. Person–Organization fit

Person–Organization fit is root in the interactionist theory of behavior (Muchinsky and Monahan, 1987) and define as “The compatibility between people and organizations” (Kristof, 1996). It means that when at least one entity meets the needs of another entity or two entities have similar basic characteristics or both, compatibility and supplementary fit between people and organizations will be realized (Kristof, 1996). Some studies believe that work values are the core factor in judging P-O fit (Kristof, 1996; Sekiguchi, 2007). The higher the level of PSM of individuals, the more likely they are to seek to join the public sector (Perry and Wise, 1990; Kim, 2018). This view is consistent with P-O fit. P-O fit thus is often used to explain the antecedents and consequences of PSM (Vandenabeele, 2007; Steijn, 2008). Bright (2007) argues that the reason why individuals with high PSM level are attracted to public organizations is that these organizations include working conditions and tasks that support their public service motivation. Individuals strongly agree with the cultural values and mission of the organization, and organizations will also choose employees who match their cultural values (Bright, 2007; Kim, 2012; Jin et al., 2018). To sum up, individuals will choose to enter the public sector due to the consistency of their values. In the process of organizational cultivation and socialization, cultural values may also become an explanatory variable of employees’ public service motivation (Moynihan and Pandey, 2007; Andrews, 2016).

### 2.2. Applicability of cross-cultural research on public service motivation

#### 2.2.1. The relationship between Confucian culture and public service motivation

The research on public service motivation originated from American society. Perry and Wise (1990) analyzed public service motivation from rational, normative and affective dimensions, and incorporated the social context variable into this framework. The process theory of public service motivation holds that public service motivation is cultivated through the process of socialization (Moynihan and Pandey, 2007), which reflects a series of values, norms, and beliefs (Perry and Hondeghem, 2008). It has been confirmed that the public service systems of various countries imply unique cultural values, and there are different modes and levels of public service motivation in different cultural fields (Anderfuhren-Biget, 2012; Gupta et al., 2020), for instance, China (Lee et al., 2020) and Republic of Korea (Kim, 2009; Lee et al., 2020) in East Asia under the guidance of Confucian culture, India with ancient civilization (Gupta et al., 2020), Iraq (Hassan and Ahmad, 2021), and Pakistan (Quratulain et al., 2019) under the dominance of Islamic culture, all of which have different public service motivations with different structures and dimensions. No matter from the perspectives of concept, structure or measurement, public service motivation has distinctive cultural characteristics.

Due to the cultural differences in public service motivation, scholars began to pay attention to Confucian culture which is the ancient and powerful cultural foundation in East Asia, and explored how Confucian values affect individual public service motivation. For example, Kim (2009) believed that due to the high homogeneity of Korean society, Koreans have a common sense of national identity and cultural identity (Macdonald and Clark, 2018). Under the influence of Confucian virtues and collectivist cultural atmosphere, Koreans tend to become civil servants and serve the public interest. Kang et al. (2017) believed that “loyalty,” “righteousness,” “benevolence,” and “relationship orientation” in Confucian values have a positive impact on normative and affective motivations. Lee et al. (2020) confirmed through the data of public sectors in China and ROK that the Confucian culture based on collectivism can affect public service motivation. Apparently, existing studies have confirmed that Confucian values are one of the antecedents influencing the public service motivation of public servants (Lee et al., 2020; Kim and Torneo, 2021).

#### 2.2.2. The Confucian culture and public service motivation in the Chinese context

It is necessary to analyze the relationship between sociocultural characteristics and public service motivation to explain the public service motivation of Chinese public servants. The public service motivation was originally put forward by western scholars to break through the shackles of the “economic man” hypothesis for explaining the action power of public servants. Perry found that the salary of the American public sectors was significantly lower than that of the private sectors, but the public sectors still attracted a large number of individuals for employment, and it was the public service motivation that made up the salary incentive gap. Although there is no huge gap between the public and non-public sectors in China in guaranteeing the individuals’ basic needs, the characteristic behaviors of voluntary contributions of Chinese public servants, such as “working voluntarily on Saturday,” “five plus two (five weekdays + weekend),” and “white plus black (daytime +nighttime),” can not be analyzed simply by “rational economic man” (Han and Sun, 2019). Besides, scholars claimed that in addition to extrinsic incentives such as material factors, these active altruistic behaviors also derive from the influence of altruistic value norms such as “virtue” and “benevolence” in traditional Confucian culture for a long time (Yang and Yang, 2021), and become the value foundation of “serving the people wholeheartedly” among Chinese public servants. Studies have shown that the working atmosphere created by the Confucian culture can promote the loyalty and perseverance of the members of the organization, which is an explanation for the overtime and self-dedication of public servants (Kim and Torneo, 2021).

Liu (2009) proposed that the public service motivation based on norms and affections in the attitude, morality and behavior of Chinese public servants can be attributed to Confucian values. Li (2014), based on the theoretical analysis of cultural orientation, mapped the “benevolence,” “righteousness,” “neutralization,” and “the greater self” in Chinese Confucian culture to Perry’s four dimensions to construct a cultural expansion scale, and verified the relationship between Confucian culture and public service motivation. Mei et al. (2021) argued that Chinese people are deeply influenced by traditional culture. For example, the values of “benevolence,” “courtesy,” and “righteousness” in Confucian culture can improve the level of public service motivation of public servants, while negative concepts such as “bureaucratic standard” hinder the development of public service motivation. It can be seen that the academia has gradually noticed that the Confucian culture is a force that affects the public service motivation, and has carried out theoretical deconstruction and practical verification in succession. However, there is still a lack of further empirical analysis between the Confucian culture and the public service motivation in the current Chinese context, and there is insufficient observation on the expanded vision of the Confucian culture. Having recognized the limitations of previous studies, this study takes the “individual traditionality” characteristics of the Chinese rooted in Confucian culture as the antecedent variable of public service motivation, and uses the survey data of 13 Chinese cities to interpret the applicability of public service motivation based on specific cultural background. Given that, hypothesis 1 is proposed:

*H1: Confucian culture has a significant effect on public service motivation.*

### 2.3. How Confucian culture affects public service motivation

Confucian classics such as *The Analects of Confucius*, *The Book of Rites*, and *Mencius* reveal that such ideas as “the whole world as one community,” “people-oriented,” “a gentleman maintains his own integrity in obscurity and endeavors to benefit the world in times of success” had a wide impact on ancient Chinese bureaucrats, and these philosophies and public service motivations had different approaches but equally satisfactory results. The value system of Confucian culture is diverse and complex, in which a variety of political, ethical and moral norms are integrated. Unlike most western theories, Confucianism has no unified theory of knowledge (Zhao, 2021). From primitive Confucianism to modern neo-Confucianism, different times, different historical backgrounds, and different interpreters endow it with different meanings through inheritance and creation (Zhang and Dong, 2021; Lu, 2022; Zhu, 2022). This study focuses more on the effect mechanism of “submission to authority” and “fatalism and defensiveness” in public service motivation. First of all, these two kinds of Chinese unique psychology are produced under the influence of traditional Chinese Confucian culture (Yang, 2004), and they widely exist in Confucian classics. For example, the Confucian system emphasizes the “Three Cardinal Guides (ruler guides subject, father guides son, and husband guides wife) and Five Constant Virtues (benevolence, righteousness, propriety, knowledge, and sincerity),” that is, the dominance of authority. In addition, the Confucian culture advocates “submission to the destiny,” and the traditional Confucianism maintains interpersonal relationships through the interpretation of “fatalism” to build a harmonious, stable, and sustainable family and society (Yang, 2004). Second, in interdisciplinary research, they are considered as part of the “traditional” Chinese psychology (Yang, 2004; Kao and Lu, 2006), and represents different connotations of Confucian values. Finally, in the relationship between Confucian culture and public service motivation, few scholars pay attention to these two dimensions. Therefore, this study selects “submission to authority” and “fatalism and defensiveness” as the dimensions to measure Confucian culture.

#### 2.3.1. The influence of submission to authority on public service motivation

Submission to authority emphasizes that in a social situation full of various roles, identities and interpersonal relationships, authority should be observed, complied with and trusted. Submission to authority actually stems from a well-known Confucian value: filial piety. At first, under the rule of filial piety, in order to show respect for parents’ seniority, children must obey their parents’ wishes and suppress their own desires to maintain a harmonious family relationship. From ancient times to the present, filial piety has been regarded as a traditional virtue by Chinese people and is highly respected. In the process of socialization, obedience to parents can easily be generalized to local officials (parents-like magistrates) through the process of pan-familism, and then extended to the emperor who has the highest ruling power so that officials and monarchs have absolute authority like fathers in people’s hearts, which also constitutes the “authoritarianism orientation” of Chinese people (Yang, 2004).

In ancient China, the traditional culture based on Confucian ethics highly respected the authority of the monarch. The worship and pursuit of status and power not only formed the “authoritarianism orientation,” but also promoted the “bureaucratic standard,” which became one of the social psychology derived from the bureaucratic political system. The worship of authority, the yearning for power, and the pride of being officials permeated all corners of ancient Chinese society, and are still influencing the present. Empirical evidence shows that China’s hierarchical culture is positively correlated with policy making-oriented PSM (Lee et al., 2020). P-O fit indicates that, compared with those who strongly desire autonomy, those who submit to authority would feel better personal organizational fit, trust the organization more and respect the leadership authority, and show more positive working attitude and motivation (De Cooman et al., 2009), thus promoting PSM. Since authoritarian leaders usually require their subordinates to fully follow their rules, they will hold them accountable if they do not obey orders (Chan et al., 2013). Therefore, from the perspective of self-protection and higher performance (Wang and Guan, 2018), submission to authority may promote the APP dimension of PSM. Submission to authority represents high power distance (Begley et al., 2002). In Confucian culture, state leaders and public sector managers have the obligation to ensure peace, prosperity and justice to make people well-being (Frederickson, 2002), while individual subjective values are usually guided by collective common culture and personal experience (Schwartz, 1992). Therefore, in the face of organizational difficulties and work contradictions, employees who uphold the values of submission to authority will give priority to the “the whole belongs to all” and “serving the people” advocated by the organizational values, and emphasize the commitment to public interests. At the same time, Confucian collectivist culture attaches great importance to harmony, reciprocity (Frederickson, 2002) and helping others (Varela et al., 2010), requires individual interests to be subordinate to collective interests, and will protect collective welfare at the cost of individual interests (Moorman and Blakely, 1995). Therefore, the more subordinate to the collective authority, the more likely it is to strengthen the COM and SS dimensions in PSM. Based on this, hypothesis 2 is proposed:

*H2: Submission to authority in Confucian culture has a significant influence on public service motivation.*

*H2.1: Submission to authority is positively correlated with public service motivation from the dimension of attraction to politics and policy making (APP).*

*H2.2: Submission to authority is positively correlated with public service motivation from the dimension of commitment to the public interest (CPI).*

*H2.3: Submission to authority is positively correlated with public service motivation from the dimension of compassion (COM).*

*H2.4: Submission to authority is positively correlated with public service motivation from the dimension of self-sacrifice (SS).*

#### 2.3.2. Influence of fatalism and defensiveness on public service motivation

Fatalism emphasizes the beliefs in fate, luck and opportunity, as well as the helplessness of external environmental factors (such as money and power), which is an external control belief (Rotter, 1966), while defensiveness emphasizes the self-serving behavior of “being wise for one’s own life” and “minding one’s own business” because of the sense of powerlessness generated by the external control tendency (Yang, 2004). Empirical studies have shown that the external control tendency will reduce individual’s willingness to help others and show selfish and aggressive behaviors, such as making people more inclined to cheat in exams (Baumeister et al., 2009). Therefore, the fatalistic and defensive attitude will urge public servants to strive for greater rights/powers to seek asylum for personal and family interests, including preference to use relationships to seek work or bribery for convenience (Yang, 2004). To sum up, the fatalistic and defensive attitude may positively affect the public service motivation based on reason and selfish APP dimensions.

Individuals who hold the fatalistic and defensive attitude tend to have external attribution tendency in their behavioral characteristics, and are easy to attribute success or failure to the predetermined “fate.” Therefore, individuals with a fatalistic and defensive attitude tend to avoid the world passively or protect themselves when they are confronted with difficulties and obstacles. Yang (2004) confirmed through research that the fatalistic and defensive attitude of Chinese people is negatively correlated with job performance and organizational input. The self-protection attitude of “more is better than less” and “nothing matters” will reduce the individual’s dedication to solving problems. In general, while promoting public servants to participate in public decision-making and strive for their own interests, they will also reduce the effort for concerning about public interests, thus negatively affecting the CPI, COM and SS dimensions of public service motivation. Based on this, hypothesis 3 is proposed:

*H3: Fatalism and defensiveness in Confucian culture has a significant influence on public service motivation.*

*H3.1: Fatalism and defensiveness is positively correlated with public service motivation from the APP dimension.*

*H3.2: Fatalism and defensiveness is negatively correlated with public service motivation from the CPI dimension.*

*H3.3: Fatalism and defensiveness is negatively correlated with public service motivation from the COM dimension.*

*H3.4: Fatalism and defensiveness is negatively correlated with public service motivation from the SS dimension.*

### 2.4. The moderating role of paternalistic leadership

The traditional Confucian culture not only shaped people’s values, but also gave birth to paternalistic leadership integrating authoritarianism, benevolence and morality (Farh and Cheng, 2000). Paternalistic leadership is based on the Confucian “family and the world” culture. It is defined as a “leadership style of strict discipline and authority, fatherly benevolence and moral integrity,” including three dimensions of authoritarianism, benevolence, and morality (Farh and Cheng, 2000), which is an important feature of the current Chinese organizations (Cheng et al., 2004). Members who comply with authoritarianism are ready to maintain this hierarchical relationship and make more efforts to safeguard the interests of leaders and the collective (Cheng et al., 2004; Shen et al., 2019). Patriarchal leadership shows the interactive relationship between Chinese leaders and subordinates, and subordinates react differently in line with different leadership styles (Cheng et al., 2004). It can be inferred that paternalistic leadership plays a moderating role in the influence of Confucian culture on public service motivation.

#### 2.4.1. The moderating effect of authoritarianism

Authoritarianism embodies the cultural characteristics of high power distance in Chinese society, and emphasizes the “superiority and inferiority” relationship between officials at upper and lower levels. Authoritarian leaders play the role of the father in the family. In order to effectively control subordinates, they maintain their unchallenged authority in the relationship between superiors and subordinates as fathers control children. The exploration on the mechanism of authoritarianism has formed two types of views. On the one hand, scholars believe that authoritarianism has a positive impact on individual organizational citizenship behavior (OCB), and it will stimulate highly traditional individuals to have a high degree of relationship recognition, obedience, and gratitude to the leadership authority. Members who comply with authoritarianism are ready to maintain this hierarchical relationship and make more efforts to safeguard the interests of leaders and the collective (Cheng et al., 2004; Shen et al., 2019). While protecting themselves from the censure of authoritarian leaders, they also strive to gain personal interests from the organizational interests. From this perspective, authoritarianism may generate positive incentives for rational public service motivation from the APP dimension.

On the other hand, a number of scholars believe that the low recognition of authoritarianism will inhibit the internal motivation of organizational members. Since authoritarianism means a distinct hierarchical relationship and power distance, in the absence of other value norms to attract individual identity, organizational members may regard authoritarian behavior as a threat to their own identity and respond with negative work attitude and resistance (Shen et al., 2019). According to the Over-justification Effect (Deci et al., 1995), the authoritarianism behavior shows strict, controlled, and even threatening external pressure, which tends to create psychological distance between public servants and leaders and organizations, preventing them from actively coping with challenges and seeking breakthroughs (Tuan, 2018). Therefore, the more authoritarianism a leader shows, the lower the internal motivation of subordinates (Chen et al., 2014) who are only willing to focus on profitable behaviors such as salary and welfare, rather than performing tasks to improve public services.

In a word, authoritarianism may be effective in seeking consistency of organizational goals, but it has a restraining effect in truly encouraging public servants to “serve the people wholeheartedly,” thus negatively affecting the public service motivation from the CPI, COM, and SS dimensions. Based on this, hypothesis 4 is proposed:

*H4: Authoritarianism plays a significant role in moderating the relationship between Confucian culture and public service motivation.*

*H4.1: Authoritarianism positively moderates the influence of Confucian culture on public service motivation from the APP dimension.*

*H4.2: Authoritarianism negatively moderates the influence of Confucian culture on public service motivation from the CPI dimension.*

*H4.3: Authoritarianism negatively moderates the influence of Confucian culture on public service motivation from the COM dimension.*

*H4.4: Authoritarianism negatively moderates the influence of Confucian culture on public service motivation from the SS dimension.*

#### 2.4.2. The moderating effect of morality

The Confucian culture places great emphasis on personal moral cultivation, which can be described as “self-cultivation, a well-managed family, and the ability to administer the state and to bring peace to the nation.” Moral leaders are highly respected and admired by Chinese employees and regarded as ideal leaders because of their integrity and concern for collective interests rather than their own interests (Niu et al., 2009). Studies have confirmed that subordinates’ perception of leaders’ moral level has a significant positive correlation with subordinates’ job satisfaction, organizational commitment, and feelings towards the organization (Davis and Rothstein, 2006). When organizational members feel that leaders are fair, honest, and exemplary, they tend to identify with and internalize leaders’ values and the collective goals they pursue, actively participate in public decision-making based on discretion, and are willing to make more personal contributions to the collective interests (Farh and Cheng, 2000), therefore, morality behavior can promote the behavior of organizational members both within and outside their roles (Chen et al., 2014). Based on this, hypothesis 5 is proposed:

*H5: Morality plays a positive role in moderating the relationship between Confucian culture and public service motivation.*

*H5.1: Morality positively moderates the influence of Confucian culture on public service motivation from the APP dimension.*

*H5.2: Morality positively moderates the influence of Confucian culture on public service motivation from the CPI dimension.*

*H5.3: Morality positively moderates the influence of Confucian culture on public service motivation from the COM dimension.*

*H5.3: Morality positively moderates the influence of Confucian culture on public service motivation from the SS dimension.*

#### 2.4.3. The moderating effect of benevolence

In the Confucian society, a kind of responsibility based on the culture of benevolence existed between the ideal superiors and subordinates, that is, the benevolence from superiors to subordinates, and the reciprocation from subordinates to superiors, demonstrating a harmonious and mutually beneficial relationship. Research has confirmed that when subordinates feel enough support from supervisors, they will improve their willingness and actions to continue working hard for the organization (Eisenberger et al., 2002). Benevolence is not only reflected in the individual concern for the work of subordinates, but also extends this concern to the overall care of their family members, trying to get along with subordinates like a family (Farh and Cheng, 2000). This also forms an affective interest bond between leaders and subordinates. In Chinese public sectors, public servants are deeply influenced by traditional Confucian cultural values such as “courtesy demands reciprocity” and “being grateful and considerate in return.” In order to repay the care, favors and benevolence given by leaders, the members of the organization will make themselves meet their leaders’ expectations and organizational goals through hard work and self-dedication (Farh and Cheng, 2000). Based on this, hypothesis 6 is proposed:

*H6: Benevolence plays a positive role in moderating the relationship between Confucian culture and public service motivation.*

*H 6.1: Benevolence plays a positive role in moderating the influence of Confucian culture on public service motivation from the APP dimension.*

*H6.2: Benevolence plays a positive role in moderating the influence of the Confucian culture on public service motivation from the CPI dimension.*

*H 6.3: Benevolence plays a positive role in moderating the influence of the Confucian culture on public service motivation from the COM dimension.*

*H 6.4: Benevolence plays a positive role in moderating the influence of the Confucian culture on public service motivation from the SS dimension.*

**FIGURE 1:**
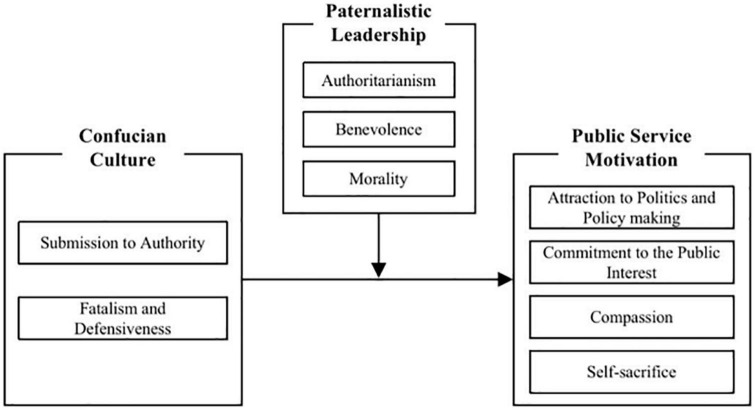
Schematic diagram of relationship among variables.

Based on the above analysis, the relationships between variables in this study are shown in Figure 1.

## 3. Research methodology, variables, and data sources

### 3.1. Research methodology

This study selected the Fixed Effect Model and the dependent variable “public service motivation” as the continuous variable. The data were collected from 12 Chinese cities. The differences in the administrative and socioeconomic environment of different cities may have an impact on the perception of individuals. Besides, individuals may encounter the problem of missing variables over time. In order to control such unmeasured heterogeneity, this study adopted the dual fixed effects model including city and time (Gormley and Matsa, 2014). The equation is as follows:


$$Y_{i\,c\,t}=\alpha_{0}+\sum \beta \,x_{i\,c\,t}+\chi_{i}+\delta_{i}+\varepsilon_{i\,c\,t}$$

*Y*_*ict*_ represents the dependent variable. α_*0*_ refers to the constant term, i is the individual serial number, c represents the city’s serial number, t represents the year. ∑β*x*_*ict*_ represents the independent variable, β is the coefficient, χ_*i*_ is the city fixed effect, *δ*_*i*_ is the time fixed effect, and *ε*_*ict*_ refers to random error.

### 3.2. Measurement of variables

The dependent variable is public service motivation. Referring to Perry’s Public Service Motivation Scale, this study set up a total of 15 items. The APP measurement included three items, which was reverse measurement. Four items were to measure the CPI, three items for the COM measurement, five items for the SS measurement, and the Cronbach’s α is 0.838.

The core independent variable is Confucian culture. Traditional Chinese psychology rooted in Confucian culture includes submission to authority, filial piety and ancestor worship, conservatism and endurance, fatalism and defensiveness, and male dominance (Yang, 2004). This study focuses on two independent variables, namely, (a) submission to authority, with Cronbach’s α being 0.712; (b) fatalism and defensiveness, with Cronbach’s α being 0.8.

The moderating variable is paternalistic leadership. The Paternalistic Leadership Scale developed by Farh and Cheng (2000) is used for the survey, and the ternary structure corresponds to the two measurement items, respectively. The Cronbach’s α is 0.76.

The above variables were all measured by the Likert 5-point scale, ranging from 1 (disagree strongly) to 5 (agree strongly). The mean value is the continuous variable.

There are two types of control variables:

1. Demographic variables. Research has confirmed that demographic factors such as gender, age, and education level will have a significant impact on individual’s public service motivation (Perry, 1997; Vandenabeele, 2007). (a) Gender. (b) Age. Perry (1997) confirmed that age is positively correlated with public service motivation, which is a continuous variable. (c) Education level. Generally speaking, the public service motivation of highly educated groups is higher than that of low educated groups (Perry, 1997; Moynihan and Pandey, 2007). (d) Official rank. Xu and Li (2020) verified that the higher the individual’s official rank, the stronger the public service motivation.
2. Sociological variables. Studies have confirmed that social and historical background factors are the antecedents of public service motivation (Vandenabeele, 2007, 2011; Perry and Hondeghem, 2008). (e) The political affiliation. In China’s public service sectors, those with the Communist Party of China (CPC) membership tend to recognize the purpose of “serving the people wholeheartedly” (Duan, 2019), showing a higher level of public service motivation. (f) Social status. Those with higher social status have higher political responsiveness (Duan, 2019), and the level of public service motivation may be more significant. Social status refers to the objective measurement method proposed by Qian and Shen (2012), and uses the official rank measurement of public servants. The economic status is measured by the 10-point scale of the local economic level, where 1 means the lowest and 10 the highest.

### 3.3. Data sources

The data used in this study were collected by the research team. The survey was conducted from June 2018 to September 2022 among public servants in 14 district-level governments in 12 cities, including Shenzhen (Nanshan District, Banan District, and Longhua District), Hangzhou, Haikou, Suzhou, Taizhou, Nanchang, Quanzhou, Wuhan, Taiyuan, Datong, Tianjin, Wuxi, and Nanjing. The survey was arranged at the district and sub-district levels with the help of local “gatekeepers,” and distributed according to the proportion of department personnel, mainly through written questionnaires and partly electronic questionnaires. It was estimated that 200 questionnaires would be distributed, and in reality150 were distributed to each city. Totally, 2,100 questionnaires were distributed, among which 1,308 questionnaires were valid, with a recovery rate of 62.29%. Constrained by objective conditions and impossible to obtain the sample data covering the whole country, the factors such as region, level, rank, and department had been considered in the sampling of this study. The survey data were of value for the exploratory study. The descriptive statistical results of effective samples are shown in Table 1. The samples covered the groups concerned by the core independent variable, involving demographic and other control variable measurement factors (gender, age, education level, rank, socioeconomic status). In general, the survey data were representative and supportive for the research hypotheses to make reasonable inference.

**TABLE 1:** Descriptive statistical results of samples.

Categorical variables	Category	Code	%
Sex	Male	0	44.44
	Female	1	55.56
Age (year)	Under 30	1	36.87
	31–50	2	48.44
	Over 51	3	14.69
Education level	Below high school	1	3.99
	Junior college	2	8.70
	Bachelor	3	65.20
	Master	4	20.98
	Doctor	5	1.79
Social status (official ranks)	Section member	1	61.31
	Unit chief level	2	11.31
	Deputy section chief/Township level	3	11.68
	Section chief/Township level	4	6.20
	Deputy county/division chief level or above	5	9.49
Continuous variables	Range of values	Average	Std. D.
Economic status	1–10	5.37	2.11
PSM-APP	1–5	2.60	1.01
PSM-CPI	1–5	3.02	0.72
PSM-COM	1–5	3.47	0.96
PSM-SS	1–5	3.38	0.79
Confu-authority	1–5	3.23	0.80
Confu-self	1–5	2.61	0.91
Leader-authority	1–5	2.96	0.85
Leader-ethics	1–5	3.42	0.90
Leader-kindness	1–5	3.39	0.89

## 4. Empirical findings

### 4.1. Benchmark model

Table 1 shows the sequence of the mean value of the four dimensions of public service motivation in the core variable: COM (3.47) > SS (3.38) > CPI (3.02) > APP (2.60), indicating that the public service motivation level based on COM of the respondents is the highest while that based on APP is the lowest, which is consistent with the research results of Kim (2009), Liu and Wang (2022), etc., that is, the East Asian public servants affected by traditional Confucian culture are not closely correlated with reason-based public service motivation. In addition, the mean value of submission to authority and fatalism and defensiveness is 3.23 and 2.61, respectively, which shows that grassroots Chinese public servants have a relatively high level of Confucian culture in terms of submission to authority while that in terms of fatalism and defensiveness is relatively general. On the whole, the cross-cultural applicability of public service motivation to grassroots Chinese public servants has been initially supported.

Table 2 presents the results of the benchmark model. First, from the regression results of Models (1)–(4), submission to authority is significantly correlated with public service motivation from the dimensions of APP (−0.254) and SS (0.179), and fatalism and defensiveness is correlated with public service motivation from the dimensions of APP (0.310), CPI (−0.079), COM (−0.086), and SS (−0.069), respectively, revealing that submission to authority and fatalism and defensiveness in the Confucian cultural values can explain the public service motivation of Chinese public servants, and the results strongly support H1 of this study.

**TABLE 2:** Confucian culture level in 12 cities.

City	Confucian-submission to authority	Confucian-fatalism and defensiveness
Shenzhen	3.11 (0.063)	2.47 (0.072)
Hangzhou	3.59 (0.061)	2.27 (0.061)
Haikou	3.21 (0.074)	2.46 (0.08)
Suzhou	3.2 (0.039)	3.04 (0.048)
Taizhou	3.2 (0.186)	3.04 (0.203)
Nanjing	3.33 (0.247)	2.74 (0.322)
Wuxi	2.91 (0.211)	2.94 (0.205)
Datong	3.44 (0.073)	2.41 (0.099)
Tianjin	3.31 (0.081)	2.51 (0.112)
Nanchang	3.68 (0.156)	2.39 (0.123)
Quanzhou	3.29 (0.11)	2.18 (0.088)
Wuhan	3.22 (0.101)	2.38 (0.097)

Second, Table 3 shows the level of Confucian culture in 12 cities. The level of Confucian culture in different cities is different. In the measurement of the level of Confucian culture, in order to overcome the limitations of subjectivity, existing studies have constructed geographic-proximity-based Confucianism variables such as the number of Confucian colleges in provinces and regions to measure the level of Confucian culture. The results show that Chinese provinces have different levels of Confucian culture (Du, 2015; Xu et al., 2019). Some research suggest that Chinese cities have regional differences with local characteristics, which are attributed to the historical imprint of Confucian culture (Obschonka et al., 2019). For example, the Confucian culture originated in Shandong (Wei et al., 2010; Obschonka et al., 2019), Shandong thus may have a high level of Confucian culture. In addition, Jiangxi is regarded as the birthplace of neo Confucianism (Walton, 2018), and the number of Confucian colleges is the largest in China (Du, 2015). Therefore, Jiangxi may also have a higher level of Confucian culture. To sum up, the empirical results of this study further verify this conclusion, that is, different cities have different levels of Confucian culture, which also explains to some extent why countries and regions with different cultural values have different levels of public service motivation.

**TABLE 3:** The regression results of the influence of Confucian culture on public service motivation.

	PSM_APP	PSM_CPI	PSM_COM	PSM_SS
	**(1)**	**(2)**	**(3)**	**(4)**
**Confucian culture**				
Submission to authority	−0.254**	0.060	0.086	0.179**
Fatalism and defensiveness	0.310***	−0.079**	−0.086*	−0.069**
**Demographic variables**				
Gender	0.229	0.024	0.013	0.037
Age (year)	−0.030	−0.003	0.008	0.007
Education level	0.056	−0.107	−0.077	−0.213*
**Sociological variables**				
Political affiliation	0.045	−0.012	0.004	−0.032
Official ranks	0.015	0.043	−0.000	−0.027
Economic status	−0.055	0.013	0.117***	0.075**
Fixed effects (city)	Yes	Yes	Yes	Yes
Fixed effects (time)	Yes	Yes	Yes	Yes
_cons	60.757	8.846	−11.350	−9.652
N	1,221	1,117	1,128	1,128
*R*-squared	0.1726	0.0637	0.1489	0.1397
Adj *R*-squared	0.1090	−0.0083	0.0834	0.0735

***p < 0.01, **p < 0.05, *p < 0.1.

Third, the regression results of Model (1) show that there is a significant negative correlation between submission to authority and APP. This result is contrary to H2.1, that is, the higher submission to authority, the lower the APP level may be. The regression coefficient (0.310) of fatalism and defensiveness is positive and significant at the level of 0.01. This result supports H3.1, indicating that the stronger the fatalism and defensiveness is, the higher the PSM level from the dimension of APP. They are more willing to engage in public affairs based on rational motivation and contribute to their own public policymaking process.

Fourth, in the Model (2)–(3), the regression coefficient of submission to authority is insignificant, indicating that submission to authority has no effect on CPI and COM, and this result fails to support H2.2 and H2.3. The regression results of Models (2)–(3) also show that fatalism inhibits the PSM level in terms of CPI and COM, which supports H3.2 and H3.3, that is, the stronger the fatalism and defensiveness is, the more likely it is to go beyond the values, moral constraints and public interests for personal and family interests, and the higher the CPI and COM levels are.

Fifth, the regression results of Model (4) show that SS is to a large extent positively affected by submission to authority. The more authority is submitted to, the stronger the willingness to make self-sacrifice. This is contrary to the prediction under H2.4. Model (4) also shows that fatalism and defensiveness has a significant negative impact on SS, confirming H3.4, that is, the stronger the fatalism and defensiveness is, the more attention is paid to personal interests, and it is not easy to show self-sacrifice behavior. In terms of control variables, the results of Models (3)–(4) show that the regression coefficient of economic status is significant at the level of 0.01 (0.117) and 0.05 (0.075), respectively, indicating that the higher the economic status is, the more likely it is to strengthen the willingness to improve public services due to the perception of difficulties of individuals at the “bottom” level, thus showing a high level affection-based public service motivation. In addition, the higher the education level, the lower the public service motivation in terms of SS.

### 4.2. The moderating effect of paternalistic leadership

The statistical results of the “moderating effect” test in Table 4 are as follows. First, the regression results of Model (11) indicated that under the moderating effect of authoritarianism, the original “main effect” of submission to authority turns to be significant, and the moderating effect (−0.162) negatively strengthens the COM level at the level of 0.05, but there is no moderating effect on the impact of fatalism and defensiveness on COM, and thus H4.3 has been partially verified. Models (5), (8), and (14) did not show significant moderating effect, and H4.1, H4.2, and H4.4 were not verified.

**TABLE 4:** The regression results of moderating effect of paternalistic leadership.

	PSM_APP			PSM_CPI		
	**(5)**	**(6)**	**(7)**	**(8)**	**(9)**	**(10)**
**Confucian culture**						
Submission to authority	−0.286	−0.601	0.060	0.319*	0.086	0.111
Fatalism and defensiveness	−0.067	0.581*	−0.079**	0.038	0.038	0.011
**Regulating variable**						
Authoritarianism* Submission to authority	0.014			−0.084		
Morality* Submission to Authority		0.117			−0.004	
Benevolence* Submission to authority			0.135			−0.016
Authoritarianism* Fatalism and defensiveness	0.103			−0.030		
Morality* Fatalism and defensiveness		−0.096			−0.033	
Benevolence* Fatalism and defensiveness			−0.197**			−0.019
**Demographic variables**						
Gender	0.224	0.216	0.201	0.031	0.024	0.029
Age (year)	−0.034	−0.026	−0.023	−0.003	−0.003	−0.003
Education level	0.052	0.032	0.080	−0.097	−0.128	−0.102
**Sociological variables**						
Political affiliation	0.069	0.036	0.055	−0.017	−0.011	−0.012
Official ranks	0.016	0.020	0.028	0.040	0.045	0.043
Economic status	−0.049	−0.058	−0.046	0.009	0.012	0.012
Fixed effects (city)	Yes	Yes	Yes	Yes	Yes	Yes
Fixed effects (time)	Yes	Yes	Yes	Yes	Yes	Yes
_cons	70.500	54.524	46.833	7.805	9.268	8.780
N	1,211	1,210	1,209	1,211	1,210	1,209
*R*-squared	0.1973	0.2083	0.2731	0.0910	0.0773	0.0655
Adj *R*-squared	0.1099	0.1221	0.1940	−0.0080	−0.0232	−0.0363
	**PSM_COM**			**PSM_SS**		
	**(11)**	**(12)**	**(13)**	**(14)**	**(15)**	**(16)**
Confucian culture						
Submission to authority	0.586**	0.504	0.382	0.392	0.355	0.702**
Fatalism and defensiveness	0.081	−0.097	0.025	0.034	−0.244	−0.186
**Regulating variable**						
Authoritarianism* Submission to authority	−0.162**			−0.069		
Morality* Submission to authority		−0.123			−0.055	
Benevolence* Submission to authority			−0.099			−0.169*
Authoritarianism* Fatalism and defensiveness	−0.073			−0.064		
Morality* Fatalism and defensiveness		−0.020			0.025	
Benevolence* Fatalism and defensiveness			−0.040			0.018
**Demographic variables**						
Gender	0.028	0.026	0.045	0.048	0.044	0.074
Age (year)	0.008	0.003	0.006	0.008	0.005	0.002
Education level	−0.060	−0.124	−0.056	−0.207	−0.216	−0.210
**Sociological variables**						
Political affiliation	−0.007	0.016	0.003	−0.041	−0.028	−0.033
Official ranks	−0.008	0.000	−0.004	−0.033	−0.028	−0.033
Economic status	0.109***	0.117***	0.108***	0.070	0.076**	0.064
Fixed effects (city)	Yes	Yes	Yes	Yes	Yes	Yes
Fixed effects (time)	Yes	Yes	Yes	Yes	Yes	Yes
_cons	−13.684	−4.002	−8.922	−12.160	−6.539	−1.668
N	1,211	1,210	1,209	1,211	1,210	1,209
*R*-squared	0.2088	0.1700	0.1732	0.1545	0.1435	0.1890
Adj *R*-squared	0.1226	0.0796	0.0832	0.0624	0.0502	0.1007

***p < 0.01, **p < 0.05, *p < 0.1.

Second, the results of Model (7) revealed that benevolence does not have a significant moderating effect on the impact of submission to authority on APP, but shows a negative moderating effect on the impact of fatalism and defensiveness on APP, which is contrary to the prediction of H6.1. In addition, the results of Model (16) confirmed that benevolence plays a negative role in moderating the influence of submission to authority on SS. The adjustment coefficient is −0.169, significant at the level of 0.1, which is contrary to H6.4. The moderating effect between the other three dimensions of public service motivation and benevolence is not shown, and thus H6.2, H6.3, and H6.4 are not supported, indicating that the moderating effect of benevolence on the relationship between Confucian culture and public service motivation is limited.

Third, the regression results of Models (6), (9), (12), and (15) demonstrated that morality does not show a significant moderating effect, and thus H4.1, H4.2, H4.3, and H4.4 are not supported. In general, paternalistic leadership can play a part of the moderating role in the influence of Confucian culture on public service motivation, and has a specific moderating effect on the four dimensions of public service motivation affected by the cultural values of submission to authority and fatalism and defensiveness, which further proves that the influence of Confucian culture on the public service motivation of Chinese public servants is different due to different paternalistic leadership styles.

## 5. Summary and discussion

### 5.1. Conclusion

Based on the perspective of cross-cultural research, this study collected data from 14 district-level government in 12 Chinese cities, and discussed the relationship between Confucian culture and public service motivation by establishing a theoretical framework. This framework involves two traditional Chinese Confucian cultures including submission to authority, and fatalism and defensiveness, respectively, testing the applicability of the four dimensions of public service motivation in China’s social and cultural background. The results confirmed that (a) Confucian culture has a differentiated influence on the four dimensions of public service motivation of Chinese grassroots public servants; (b) The paternalistic leadership nurtured by traditional Chinese Confucian culture partially moderates the relationship between Confucian culture and different dimensions of public service motivation.

First, this study preliminarily confirmed that Confucian culture constitutes an important cultural value orientation of the public service motivation of grassroots Chinese public servants (H1 is supported). Moreover, different cities have different levels of Confucian culture, which is related to social history. Submission to authority has a significant impact on the PSM level (H2 is partially supported). In contrast to hypothesis H2.1, the empirical results show that submission to authority inhibits the APP level. This may be because a high degree of respect for hierarchy has a negative impact on the desire to participate in decision-making, which often leads to employees’ lack of initiative in participating in decision-making (Wang et al., 2005) and creativity (Yuan and Zhou, 2015). This result may be point that a high degree of submission to hierarchy has a negative impact on the desire to participate in decision-making, which often leads to the lack of initiative of employees in participating in decision-making (Wang et al., 2005) and creativity (Yuan and Zhou, 2015). In other words, a high degree of submission to hierarchy and authority will encourage grassroots public servants to accept and follow the ideas of leaders and adopt a conservative attitude. People who at high power distance usually think that there is little value in trying to influence decisions (Begley et al., 2002). In order to maintain a harmonious relationship, they are more willing to maintain the current system order and operating rules, and give up the challenge of participating in public decision-making (Hofstede, 1980).

In addition, the empirical results are different from what we expected: the more submission to authority, the more likely individuals are to make self-dedication. This may attribute to the social respect concept of abiding by the collective interests and “the Great Way is for the sake of the public” implied in Confucian collectivism (Han and Sun, 2022). The consistent goal of national, social, organizational leaders and other authorities to improve public services helps public servants break away from external motivation and turn to the autonomous motivation of “serving the people makes me happy” (Han and Sun, 2022). Reciprocity and kindness are characteristics of highly collectivistic environment (Schwartz, 1994). Sacrificing the “individual benefit” for the sake of “great righteousness” is a virtue as well as a respectable attitude. Therefore, the higher the level of submission to authority, the stronger the public servants’ commit to “serving the people” selflessly, and the higher the SS level of sacrificing personal interests to the collective interests.

Second, the effect of Confucian culture on public service motivation is not consistent. The more fatalism and defensiveness, the higher the APP level of public service motivation, and the lower the CPI, COM, and SS levels (H3 is partially supported), revealing that when facing with difficulties, setbacks, or conflicts of interest, individuals with fatalistic and defensive attitudes tend to be passive and self-interested under the influence of external control beliefs, give priority to personal interests, and refuse to make contributions, which will reduce the normative and affective public service motivation while improving the rational public service motivation.

Third, a new mechanism is proposed between Confucian culture and public service motivation by adding paternalistic leadership as a moderator. However, the current study found that the moderating effect of paternalistic leadership is limited (H4 and H6 are partially supported). Specifically, authoritarianism only plays a negative role in moderating the influence of submission to authority on COM among the influence of Confucian culture on public service motivation. The stronger the individuals’ feeling about authoritarianism, the more likely they are to lower the COM level of public service motivation because of submission to authority. This reveals that authoritarian behavior will strengthen subordinates who originally respect authority to recognize and follow authoritarian leaders. However, due to the destruction of authoritarian behavior on social affections and interpersonal needs (Chen et al., 2014), authoritarian behavior reduces the subordinates’ organizational citizenship behavior (Cheng et al., 2002) and the initiative to participate in altruistic public services (Tuan, 2018), leading to their PSM perception breaks from the COM dimension.

Benevolence plays a negative role in moderating the influence of submission to authority on SS. It may be that benevolence and care of benevolent leaders have established an affective bond between subordinates (Chen et al., 2014; Tuan, 2018), which makes subordinates feel intimate distance and smaller power distance, and thus highly traditional individuals reduce their fear and obedience to authority, and the role of the authority will be weakened in urging organizational members to make self-sacrifice and its accountability effect on work performance accordingly. Benevolent leaders will also reduce the impact of fatalistic and defensive attitude on APP, implying that benevolence may reduce the public servants motivation for private interests and increase their positive actions for the sake of collective interests by means of personal care, affective care, tolerance, and understanding.

Furthermore, the results also confirmed that morality may not have a moderating role in the effect of Confucian culture on public service motivation, implying that Confucian culture, embodied in different roles and identities (such as leaders and subordinates), plays an interactive role in influencing public service motivation.

The current study is an attempt of localized research, breaking through the limitations of previous research on Confucian culture, paternalistic leadership, and public service motivation confined to the holistic perspective, and taking an exploratory step in the in-depth, personalized and decomposed research on incentives for public servants. In brief, our research findings provide both theoretical and practical implications. First of all, this study has developed an innovative theoretical framework. Through empirical data, it is proved that the Confucian culture rooted in the social and historical process is an antecedent variable that affects the public service motivation of grassroots Chinese public servants, and has a positive and inhibitory effect on different dimensions of public service motivation. It not only expands the cross-cultural applicability of the theory of public service motivation in non-western countries, but also supplements the evidence of research on public service motivation in East Asian countries, and suggests that more background variables such as traditional cultural values need to be examined in public service motivation.

In practice, this study also provides meaningful insights into the personnel management of Chinese public organizations. Considering that dereliction of duty in Chinese public sectors occurs from time to time, the traditional incentive mechanism for public servants becomes inefficient and the overall morale of public organizations has been frustrated (Duan and Chen, 2021), how can we recruit employees who have an attitude of serving the public interest rather than just for personal interests into the organization? How to constantly encourage public servants to continuously engage in public services? It is necessary for the organizations to consider the importance of specific cultural values for organizational culture and personal value orientation. Moreover, the organizations should pay attention to, detect and determine which cultural values can motivate public servants to improve their public service motivation. Meet the specific needs of inducement motivation, thus enhancing organizational citizenship behavior (Zhou et al., 2023), so as to enhance organizational citizenship behavior and maintain the service consciousness of “serving the people wholeheartedly”, and recruit and cultivate public employees who are consistent with the organizational culture and organizational goals. These measures may help to improve the organizational performance of the public sectors and improve public services.

### 5.2. Limitations and future research prospects

Although this study has made new findings on the relationship between Confucian culture and public service motivation, there are still limitations. First of all, we have selected samples from 12 Chinese cities, however, due to China’s huge population, vast territory, long historical background, and differences in urban regional culture, the cross-sectional data and the sample inference may represent some cities, rather than the entire grass-roots civil servants in China. Secondly, our exploration of Confucian culture is limited. The system of Confucian culture is complex and diverse. Due to space constraints, we only selected two dimensions for the measurement, trying to preliminarily verify the relationship between Confucian culture and public service motivation. Future research can pay more attention to other dimensions of Confucian culture to further build a complete framework; though the measurement scales for public service motivation in the academic community are rich, it is necessary to continuously develop a universal scale that is more fit for the Chinese situation and reflects cultural utility to enhance the explanatory power of the results and the applicability of cross-cultural scenarios. Last, from the P-O fit and the interactionist perspective, due to the complementarity and consistency between people and organizations (Muchinsky and Monahan, 1987; Kristof, 1996), individuals will maintain the organizational culture in order to maintain consistency with the organization, while employees with high public service motivation are usually reluctant to leave their work organizations (Bright, 2007). Therefore, these employees with higher levels of public service motivation will gather together to gradually form an organization’s macro background and cultural atmosphere until people and the organization are complete with each other (Bright, 2007). In other words, organizations with strong public service motivation may form a collective cultural atmosphere, and there may be endogenous problems of reverse causation. However, this study believes that SDT, P-O fit, and PSM process theory still largely support that cultural values are the antecedents of public service motivation. On the one hand, compared with PSM, the historical and cultural values of a country as a variable are more difficult to change, and the cultural background profoundly affects the socialization of individuals (House et al., 2004). PSM is root in the process of socialization (Perry, 1997, 2000; Moynihan and Pandey, 2007; Perry and Hondeghem, 2008; Corduneanu et al., 2020), and cultural values have been proved to be the pre-factor affecting the level of PSM (Anderfuhren-Biget, 2012; Vandenabeele, 2014; Lee et al., 2020; Kim and Torneo, 2021). On the other hand, existing studies argue that employees’ values were formed before they entered the organization (Perry, 1997; Ritz and Brewer, 2013), and the impact of the work environment is secondary to the process of socialization (Ritz and Brewer, 2013). Of course, the two-way causal problem between the two still needs to be considered, especially the limitations brought by cross-sectional data. Our future’s research will conduct feasible experimental design or quasi experimental design to strictly infer the effect mechanism of Confucian culture on public service motivation, and expand the research samples in the central and western regions in China to improve the external validity of inference.

## Data availability statement

The original contributions presented in this study are included in this article/supplementary material, further inquiries can be directed to the corresponding author.

## Author contributions

CD contributed to the empirical work, analysis of the results, and writing of the first draft. JJ, CZ, and YL supported the total work of CD. JJ, CZ, and YL revised and supervised the manuscript. All authors contributed to the article and approved the submitted version.
